# Cellulose Nanofiber Paper as an Ultra Flexible Nonvolatile Memory

**DOI:** 10.1038/srep05532

**Published:** 2014-07-02

**Authors:** Kazuki Nagashima, Hirotaka Koga, Umberto Celano, Fuwei Zhuge, Masaki Kanai, Sakon Rahong, Gang Meng, Yong He, Jo De Boeck, Malgorzata Jurczak, Wilfried Vandervorst, Takuya Kitaoka, Masaya Nogi, Takeshi Yanagida

**Affiliations:** 1 The Institute of Scientific and Industrial Research, Osaka University, 8-1 Mihogaoka Ibaraki, Osaka, 567-0047, Japan; 2 IMEC, Kapeldreef 75, B-3001 Heverlee (Leuven), Belgium; 3 KU Leuven, Department of Physics and Astronomy (IKS), Celestijnenlaan 200D, 3001 Leuven, Belgium; 4 Department of Agro-environmental Sciences, Graduate School of Bioresource and Bioenvironmental Sciences, Kyushu University, Fukuoka, 812-8581, Japan

## Abstract

On the development of flexible electronics, a highly flexible nonvolatile memory, which is an important circuit component for the portability, is necessary. However, the flexibility of existing nonvolatile memory has been limited, *e.g*. the smallest radius into which can be bent has been millimeters range, due to the difficulty in maintaining memory properties while bending. Here we propose the ultra flexible resistive nonvolatile memory using Ag-decorated cellulose nanofiber paper (CNP). The Ag-decorated CNP devices showed the stable nonvolatile memory effects with 6 orders of ON/OFF resistance ratio and the small standard deviation of switching voltage distribution. The memory performance of CNP devices can be maintained without any degradation when being bent down to the radius of 350 μm, which is the smallest value compared to those of existing any flexible nonvolatile memories. Thus the present device using abundant and mechanically flexible CNP offers a highly flexible nonvolatile memory for portable flexible electronics.

Flexible electronics is an emerging research field in electronics, which has been intensively investigated in last decade^1^. Novel devices such as rollable display, conformable sensor, biodegradable electronics and other flexible electronic applications have been demonstrated and they have been achieved by utilizing mechanically flexible electronic circuit components^1^,^2^,^3^,^4^,^5^,^6^,^7^,^8^,^9^,^10^,^11^,^12^. For next-generation flexible electronics, it is strongly desired to develop highly flexible circuit components, which can be tightly rolled and bent around sharp edges without degradation of the electric performance. Therefore, recent studies on flexible electronics have focused on developing highly flexible circuit components^8^,^9^,^10^,^11^,^12^. For example, Sekitani *et al.* reported flexible organic thin film transistor (TFT) with the bending radius of 100 μm^8^. The performance of TFT was maintained as long as the gate insulator withstood the bending stress.

Nonvolatile memory, which is an essential component for portable and self-standing electronics, is an important flexible circuit component^13^,^14^,^15^,^16^,^17^,^18^,^19^,^20^,^21^,^22^,^23^,^24^,^25^,^26^,^27^,^28^,^29^,^30^,^31^,^32^,^33^,^34^,^35^,^36^,^37^,^38^,^39^,^40^. Previously, several types of flexible nonvolatile memory such as flexible flash memory^13^,^14^,^15^,^16^,^17^, flexible ferroelectric memory^18^,^19^ and flexible resistive memory^20^,^21^,^22^,^23^,^24^,^25^,^26^,^27^,^28^,^29^,^30^,^31^,^32^,^33^,^34^,^35^,^36^,^37^,^38^ have been demonstrated. In these previous flexible nonvolatile memories, the smallest radius into which the memory devices can be bent has been limited to several millimeters due to the strain-induced degradation of memory properties^40^. Needless to say, further flexibility of nonvolatile memory beyond the current limitation is desired toward highly flexible and portable electronics.

Here we demonstrate an Ag-decorated cellulose nanofiber paper (CNP) as ultra flexible nonvolatile resistive memory^41^,^42^,^43^,^44^. The cellulose nanofibers, which are renewable and the most abundant biomass on earth, have outstanding properties including a high tensile strength (2–6 GPa) and a low thermal expansion coefficient (6 ppm/K)^45^,^46^. CNP has been applied to electronic device as a substrate and reinforcing agent for conductive sheets of carbon nanotubes^12^,^47^,^48^,^49^,^50^,^51^,^52^,^53^, however none of previous investigations has utilized CNP as memory devices to store electrical information. This study shows that the resistance of Ag-decorated CNP can be electrically switched with 6 orders of ON/OFF resistance ratio, and the resistance change can be utilized as nonvolatile memory effect. In addition, we found the excellent robustness of mechanical and electrical properties of the present CNP memory devices even for the bending radius of down to 350 μm, which is the smallest value so far reported for flexible nonvolatile memories.

## Results

**Figure 1:**
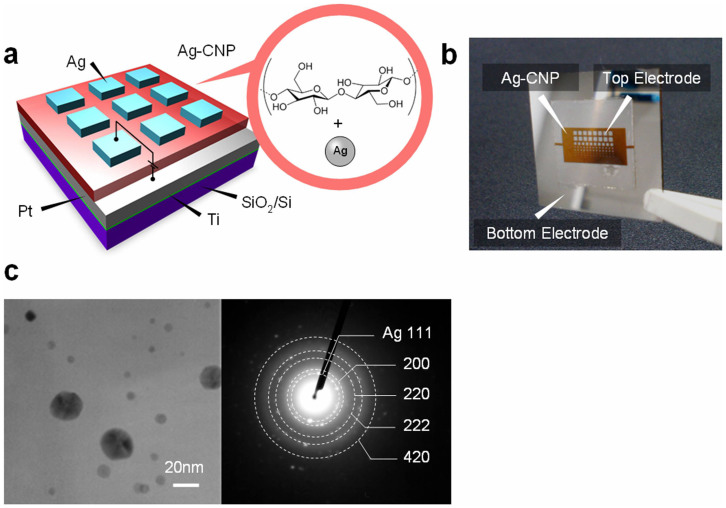
(a) Schematic illustration and (b) photograph of the Ag-decorated CNP device. (c) Cross-sectional TEM image and SAED pattern of the Ag-decorated CNP.

**Figure 2:**
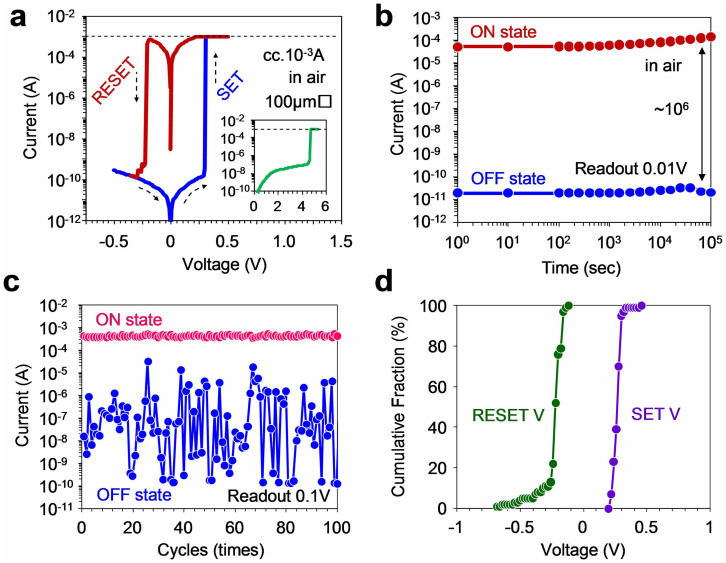
(a) *I-V* characteristics of the Ag-decorated CNP device. Current compliance of 10^−3^ A was applied. Inset shows the initial forming process. (b) Data retention and (c) switching endurance of the Ag-decorated CNP device. Readout voltages were 0.01 V for retention and 0.1 V for endurance, respectively. (d) Statistical distribution data of operation voltages for SET and RESET processes. The data is analyzed by continuous 100 switches.

The memory effect in Ag-decorated CNP device was firstly examined by utilizing devices fabricated onto the rigid Si substrate before examining its flexibility. Figure 1 (a) and (b) show the schematic illustration and photograph of the Ag-decorated CNP memory device on Si substrate. In this study, we utilized 2,2,6,6-tetramethylpiperidine-1-oxyl (TEMPO)-oxidized cellulose nanofibers of 3–4 nm width^52^,^53^,^54^,^55^ as the base matrix of CNP. The CNP layer was fabricated by drop casting of the cellulose nanofibers slurry onto Pt/Ti/SiO_2_/Si substrate and drying at 40°C for 24 hrs. The Ag nanoparticles were in situ synthesized onto the cellulose nanofibers prior to the fabrication of CNP (see the details in Method section and Supplementary Information S1). The thickness of Ag-decorated CNP can be readily controlled by varying the amount of cellulose nanofibers slurry. Then Ag electrodes of sizes ranged from 50 × 50 μm^2^ to 500 × 500 μm^2^ were deposited onto the Ag-decorated CNP device. Figure 1 (c) shows a cross-sectional TEM image of the device. Although the individual cellulose nanofibers were not visible due to the densely-packed CNP structure, the nanoparticles were observable and the size was ranged from 1 nm to 30 nm. Selected area electron diffraction (SAED) pattern identified that these nanoparticles were composed of Ag. Figure 2 (a) shows the typical current-voltage (*I-V*) data of Ag-decorated CNP device. The measurements were performed at room temperature in atmospheric condition. The thickness of the Ag-decorated CNP was ca. 2.9 μm. The Ag-decorated CNP was sandwiched by Ag top electrode and Pt bottom electrode. As shown in the inset of figure 2 (a), initially we performed the forming process with the current compliance of 10^−3^ A, which is a preparative process for resistive switching memory^56^. The applied voltage was controlled by the Ag top electrode, whereas the Pt bottom electrode was grounded. When we increased the voltage, the current abruptly increased at 4.7 V. After the forming process, the resistance state changed from insulative pristine state to conductive ON state. Then we performed so-called RESET process by applying negative voltage, which changed the resistance state back to insulative OFF state. Subsequently, ON state was recreated by applying positive voltage, which is so-called SET process. The stability of resistive switching in Ag-decorated CNP devices was confirmed by retention and endurance measurement. Figure 2 (b) and (c) show the data retention and the switching endurance of Ag-decorated CNP device. The readout voltages were 0.01 V for retention and 0.1 V for endurance, respectively. Both of the ON and OFF states were maintained for 10^5^ s with 6 orders of ON/OFF resistance ratio, indicating the nonvolatility of the present device. The endurance was confirmed at least up to 100 switches. The distribution of resistances in endurance data was caused by the small SET voltages of Ag-decorated CNP devices and should be improved by optimizing the thickness and concentration of Ag nanoparticles in CNP devices. Figure 2 (d) shows the distribution of the applied voltages required for SET and RESET processes. The data were taken from 100 continuous switches. The SET voltage is defined as a voltage when the current reaches to the current compliance value-10^−3^ A during SET process, while the RESET voltage is defined as the voltage when the current starts to drop during RESET process. The average voltage values required for SET and RESET processes were +0.28 V and −0.22 V, respectively. The standard deviations for SET and RESET voltages were 0.26 V and 0.56 V, respectively, indicating the small variation of switching voltages for memory operation. Thus, these results highlight that the present Ag-decorated CNP devices exhibit reliable nonvolatile memory effects with unambiguous memory properties including retention, endurance, ON/OFF resistance ratio and small statistical variations of operation voltage.

**Figure 3:**
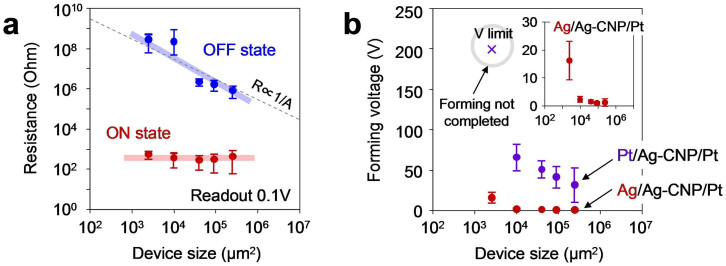
(a) The device size dependence on the ON and OFF state resistances. The Ag/Ag-decorated CNP/Pt device structure was employed. The device size was varied from 50 × 50 μm^2^ to 500 × 500 μm^2^. The readout voltage is 0.1 V. (b) The material dependece of top electrode on the forming voltage. In this experiment, we utilized Ag and Pt as the top electrodes and the device sizes ranged from 50 × 50 μm^2^ to 500 × 500 μm^2^ were examined.

Here we discuss what governs the resistive switching in the present Ag-decorated CNP devices. First, we examined the area dependence of ON and OFF state resistances to clarify the spatial inhomogeneity of electrical conduction within matrix^57^. As shown in Figure 3 (a), the OFF state resistance increased with decreasing the device size, whereas the ON state resistance did not show the size dependence. Considering previous implications, the present data infers the presence of local conduction paths within an insulating matrix^56^,^57^. Within the framework of conventional electrochemical metallization mechanism, the resistive switching can be interpreted in terms of the formation and rupture of Ag conductive filaments^42^,^43^,^44^. Ag are ionized near the positively biased electrode, and then the Ag^+^ ions migrate and diffuse into CNP layer toward negatively charged Pt counter electrode. When the Ag^+^ ions reach to the Pt electrode, the Ag^+^ ions become Ag by receiving the electrons from the Pt electrode. Such successive metallization process creates the Ag conductive filaments between the Pt electrode and the Ag electrode. The rupture of Ag filaments occurs through the ionization and the migration of Ag^+^ ions under the opposite electric field polarity. In such switching model, the ionization and migration of Ag top electrode might be critical for the resistive switching phenomena. Experimentally, the forming voltages for devices with the Ag top electrodes was found to be much lower than that for devices with Pt top electrodes, as shown in Figure 3(b). In addition, the resistive switching of Ag top electrode device was confirmed to be much more stable than that of Pt top electrode devices (Supplementary Information S2–3). These results indicate the critical role of Ag top electrode for the nonvolatile memory effect in CNP device. More details can be seen in Supplementary Information. Thus, the experimental results highlight that the model based on the formation and rupture of Ag conductive filaments is plausible for the resistive switching in the Ag-decorated CNP device (Supplementary Information S4).

Next we discuss the switching voltage value required for the initial forming process of the Ag-decorated CNP devices (V_forming_ = 4.7 V in Figure 2(a)). The calculated electric field intensity for the Ag-decorated CNP device is ca. 1.6 × 10^−2^ MV/cm, which is much lower than that for conventional resistive memory devices (0.6–1.2 MV/cm)^58^. Since the presence of metal nanoparticles is well known to enhance the electric field intensity around the nanoparticles^59^,^60^,^61^, such effect might explain the lower forming electric field intensity for the Ag-decorated CNP device. We performed the electric field simulation in the presence of metal nanoparticles, as shown in Supplementary Information S5. For qualitative understanding and simplicity for simulation, we assumed the simple series alignment of the nanoparticles to calculate the spatial distribution of electric field. The electric field intensity increases several times by introducing the metal nanoparticles, because of 1) the enhancement of electric field around the nanoparticles' edge and 2) the reduction of working distance. Since the actual forming electric field intensity for the Ag-decorated CNP device was 2 orders lower than that for the conventional resistive memory devices, the electric field enhancement effect alone cannot explain the present experimental results in this study. Although we do not have the direct experimental evidence to explain the discrepancy, we speculate that the bonding weakness between cellulose nanofibers and the water adsorbed on Ag-decorated CNP might lower the forming voltage via increasing the ion mobility of Ag^+^ ions within CNP layer. Since the cellulose nanofibers are weakly connected by hydrogen bonds and/or van der Waals forces, the cellulose nanofibers can be easily disconnected by external stimuli. In addition, the ions can stably exist and easily move in water^62^,^63^. If the forming process of Ag-decorated CNP device is related to the migration of Ag^+^ ions, the presence of water crucially affects the forming voltage. In fact, the water is known to adsorb on the surface of cellulose nanofibers due to the presence of OH bonds^64^. Although the further investigations should be undertaken to directly identify the exact origin of lowering the forming voltage, our experimental results highlight that the present Ag-decorated CNP devices have the advantages for memory operations working at low voltage range even for micrometer-scale thickness.

**Figure 4:**
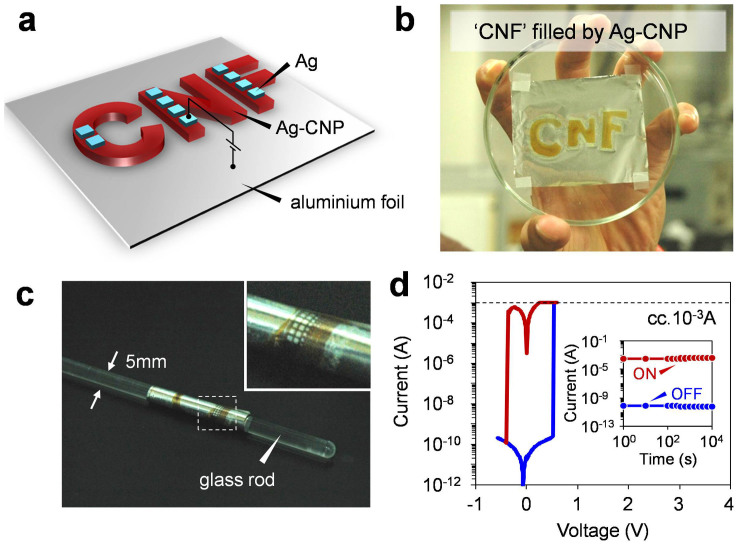
(a) Schematic illustration and (b) photograph of the Ag-decorated CNP devices on aluminium foil. (c) Photograph of the Ag-decorated CNP device wrapped around the glass rod (φ = 5 mm). Inset shows the magnified image near the device stack. (d) *I-V* characteristics of the Ag-decorated CNP device on aluminium foil (curvature radius *r* = ∞). Inset shows data retention taken at 0.1 V at room temperature in atmospheric condition.

**Figure 5:**
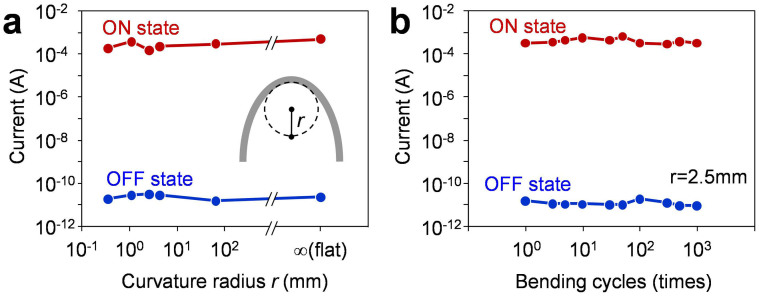
(a) ON and OFF state currents as a function of curvature radius. The data is collected from *I-V* curves performed at each curvature radius. (b) ON and OFF state currents as a function of bending cycles. Curvature radius of 2.5 mm is utilized. The data is collected from *I-V* curves performed at each bending cycles.

**Figure 6:**
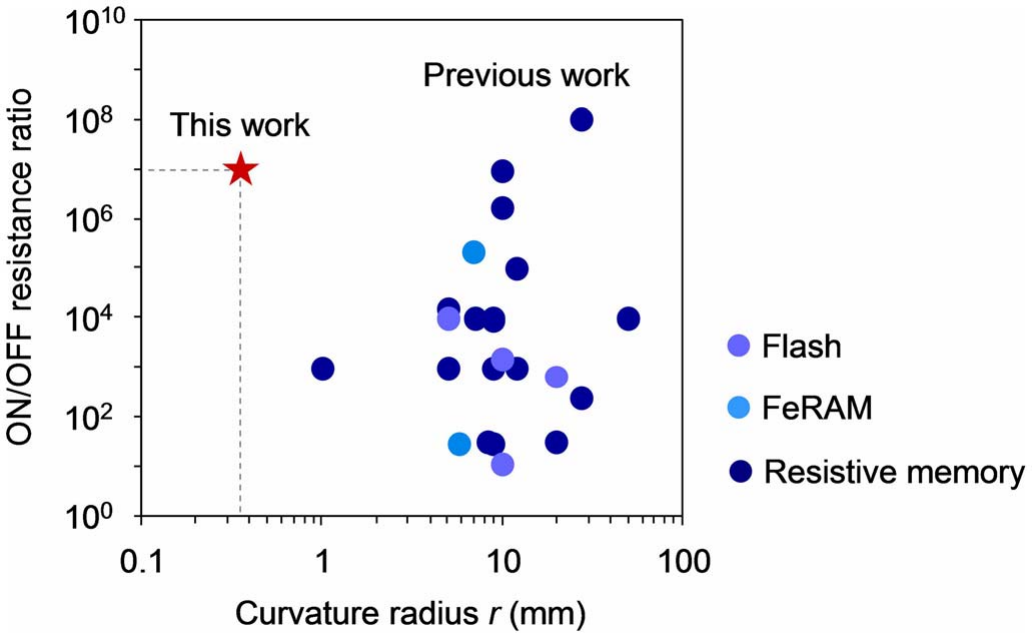
Flexible properties on nonvolatile memory devices as functions of ON/OFF resistance ratio and curvature radius. Several types of flexible nonvolatile memories including flexible flash memory^13^,^14^,^15^,^16^, flexible FeRAM^18^,^19^ and flexible resistive memory^20^,^21^,^22^,^23^,^24^,^25^,^26^,^27^,^28^,^29^,^30^,^31^,^32^,^33^,^34^,^35^,^36^,^37^,^38^ are shown.

Here we examine the flexibility of Ag-decorated CNP devices. We utilized an aluminium foil as flexible substrate. Figure 4 (a) and (b) show the schematic illustration and the photograph of Ag-decorated CNP device on aluminium foil substrate. The Ag-decorated CNP was selectively deposited onto the aluminium foil substrate by chemically modifying the substrate surface (see details in Method section and Supplementary Information S6). The bending test was performed by wrapping the Ag-decorated CNP devices around various column-shaped staffs. Figure 4 (c) shows the typical photograph of the Ag-decorated CNP device wrapped around the glass rod (φ = 5 mm). The thicknesses of Ag-decorated CNP and aluminium foil were 560 nm and 12 μm, respectively. The device can be flexibly bent, and any cracks or exfoliations were not observed even the device was bent down to the radius of 350 μm (see Supplementary Information S7). Figure 4 (d) shows the typical *I-V* curve of the Ag-decorated CNP device on aluminium foil. The aluminium foil substrate was used as the bottom electrode since the aluminium foil can maintain the electrical conductivity even bending. The clear resistive switching events and good retention were confirmed for the present aluminium foil based memory devices. Next we examine the flexibility of the present Ag-decorated CNP memory devices. Figure 5 (a) shows the ON and OFF state currents as a function of curvature radius *r*. The data was collected from the *I-V* curves performed at each curvature radius. The readout voltage was 0.1 V. Remarkably, both of the ON and OFF state currents were stably kept to be almost constant without any degradations even for *r* = 350 μm. In addition, we performed the bending cycle measurement of the Ag-decorated CNP device. Figure 5 (b) shows the ON and OFF state currents as a function of bending cycles. The curvature radius *r* and the readout voltage were 2.5 mm and 0.1 V, respectively. The data were collected from the *I-V* curves performed after the bending cycles. No significant changes for both ON and OFF state currents were observed up to 1000 cycles, indicating the excellent stability of the Ag-decorated CNP devices for the bending cycles. Now we will compare the flexible memory performance of Ag-decorated CNP device with the recent studies on flexible nonvolatile memories as functions of the ON/OFF resistance ratio and the curvature radius in Figure 6. The data contains several types of nonvolatile memory devices including flash memory^13^,^14^,^15^,^16^, FeRAM^18^,^19^ and resistive memory^20^,^21^,^22^,^23^,^24^,^25^,^26^,^27^,^28^,^29^,^30^,^31^,^32^,^33^,^34^,^35^,^36^,^37^,^38^ and several types of material including graphene, other organic materials and oxides. Our Ag-decorated CNP device shows superior flexible memory performance to any existing flexible nonvolatile memories in terms of the flexibility and the ON/OFF resistance ratio.

We discuss what produces the flexibility of the Ag-decorated CNP devices. It is noted that individual cellulose nanofiber is not flexible, *e.g.* the Young's modulus is about 145 GPa^65^. In addition, the Ag-decorated CNP utilized in this study was close packed and the space between nanofibers was not observable at nanometer scale in the TEM image of figure 1 (c). Therefore, the degree of spatial freedom for the deformation of cellulose nanofibers must be much lower than that for the conventional paper^46^,^66^. In respect to the mechanics of materials, the following two factors seem to be responsible for the flexibility of the Ag-decorated CNP devices. They are 1) the small width of cellulose nanofiber component and 2) the network structure in CNP. Considering the width of individual cellulose nanofiber- 4 nm, the maximum strain for each cellulose nanofiber can be estimated to be 0.0006% for the curvature radius of 350 μm, which is too small value to shear the cellulose nanofiber. Although the strain of 560 nm thick sheet on 12 μm thick aluminium foil reaches to 1.79% at the curvature radius of 350 μm, the substantial strain for the CNP with the fiber network structure is supposed to be smaller. Based on the Cox model, the load applied to the fiber network structure is mitigated by the random orientation of fibers, resulting in the reduction of Young's modulus of the network structure^67^. Such effect is experimentally confirmed by Sehaqui *et al.*; the Young's modulus of CNP decreased and the critical strain for breaking increased as the degree of orientation of cellulose nanofibers decreases, indicating the high flexibility of CNP with randomly oriented cellulose nanofibers network^68^. Remarkably, the critical strain for breaking of randomly oriented CNP reached to 5.26%^68^, which is much higher value than the strain applied in this study. Thus these two factors enhance the flexibility of Ag-decorated CNP devices although enough space is not secured between nanofibers.

## Discussion

In summary, we demonstrated the Ag-decorated CNP as ultra flexible electronic information storage. The Ag-decorated CNP memory device showed the nonvolatile resistive switching with 6 orders of ON/OFF resistance ratio and small variation of operation voltage. In addition, the Ag-decorated CNP devices showed the great mechanical flexibility without degradation of memory performance (bending radius of 350 μm), which was the smallest value so far reported for existing any flexible nonvolatile memory devices. Although cellulose nanofibers, which are main component of CNP and a raw material of paper, have been utilized as an ink-written information-transfer medium, these results highlight the potential use of cellulose nanofibers as functional electric information storage.

## Methods

### Synthesis and characterization of Ag-decorated cellulose nanofibers

Cellulose nanofibers with width 3–4 nm (COONa content: 1.2 mmol g^−1^) were prepared from bleached softwood kraft pulp, using the 2,2,6,6-tetramethylpiperidine-1-oxyl (TEMPO)-mediated oxidation system^50^. Then, the surfaces of cellulose nanofibers were oxidized by sodium periodate (NaIO_4_) to introduce aldehyde groups, as follows. The aqueous dispersion of cellulose nanofibers (1 wt%, 50 mL) was mixed with the aqueous solution of NaIO_4_ (12 mM, 50 mL) and then stirred at room temperature for 48 hrs. The as-treated cellulose nanofibers were washed with a mixture of 70/30 (v/v) ethanol/water (100 mL) five times, followed by removal of ethanol at room temperature under reduced pressure and dilution with deionized water. For the decoration of cellulose nanofibers with Ag nanoparticles, silver ammonia aqueous solution was prepared by adding silver nitrate (0.59 M, 2 mL) aqueous solution to ammonia aqueous solution (1 M, 2 mL), followed by dilution with deionized water (96 mL). Subsequently, the aqueous suspension of the NaIO_4_-treated cellulose nanofibers (0.1 wt%, 16 mL) was mixed with polyvinylpyrrolidone aqueous solution (1 wt%, 28 mL) and the silver ammonia aqueous solution (56 mL), in that order. The resulting mixture was stirred at room temperature for 3 hrs to form the Ag-decorated cellulose nanofibers.

The width and the microstructure of fabricated Ag-decorated cellulose nanofibers were evaluated by transmission electron microscopy (TEM; JEOL JEM 3000F) at an accelerating voltage of 300 kV. A TEM specimen was prepared by diluting the Ag-decorated cellulose nanofibers solution (cellulose nanofibers content: 0.016 wt%) by ethanol with 1:500 volume ratio and then dropping the suspension onto a microgrid.

### Fabrication and characterization of Ag-decorated CNP devices

To fabricate the Ag-decorated CNP, the Ag-decorated cellulose nanofibers solution was dropped on Pt/Ti/SiO_2_/Si(100) substrate or aluminium foil and then dried up at 40°C for 24 hrs. The thickness of Ag-decorated CNP can be controlled via the amount of Ag-decorated cellulose nanofibers solution and the spreading area. Prior to the Ag-decorated CNP deposition, Ti adhesion layer and Pt bottom electrode were sputtered on SiO_2_/Si(100) substrate with the thickness of 5 nm and 100 nm, respectively. After the Ag-decorated CNP deposition, Ag or Pt top electrode was fabricated with varying the size ranged from 50 × 50 μm^2^ to 500 × 500 μm^2^ by means of the metal mask. The microstructure, composition and crystal structure of the Ag-decorated CNP were evaluated by TEM equipped with energy dispersive electron spectroscopy (EDS). Cross-sectional TEM specimen was prepared by milling the Ag-decorated CNP device. The structural change for bending experiments was evaluated by optical microscopy equipped with digital CCD camera (OLYMPUS; BXFM-DP21). Transport properties of the devices were characterized using a semiconductor parameter analyser (Keithley 4200SCS). *I-V* curve, retention and bending experiments were performed at room temperature and in atmospheric condition.

### Hydrophilic/hydrophobic pattern preparation for selective Ag-decorated CNP deposition

To create the hydrophilic/hydrophobic pattern, several techniques including photolithography and 30 kV electron beam (EB) lithography were utilized. Prior to the lithography, 5 nm thick Ti adhesion layer and 100 nm thick Pt bottom electrode were deposited onto glass substrate. For the photolithography, AZ5206E photo resist (AZ electronic materials) was spin coated at 5500 rpm for 60 s and UV light was irradiated using Xe lamp. For the EB lithography, ZEP520A-7 (ZEON Chemicals) was used as EB resist. After the lithography process, SiO_2_ was deposited on the exposed surface area, which is used as a reactive layer for hydrophobic self-assembly monolayer (SAM). Then the substrate was immersed into trichloro-(*1H, 1H, 2H, 2H* perfluorooctyl)-silane (so-called FDTS) 1 vol% solution in perfluorooctane for 2 hrs, followed by rinsing with perfluorooctane. Subsequently, the resist film was lifted-off from the substrate by dipping in *N,N*-dimethylformamide (DMF). After the lift-off process, FDTS is absorbed only onto the SiO_2_ surface. Since the surface of Pt was hydrophilic and FDTS was hydrophobic, finally we obtained the hydrophilic/hydrophobic surface pattern. For arbitrary shaped hydrophobic pattern by hand-drawing process, we utilized a plastic pencil (coupy pencil; SAKURA Color Product Corp.). Since the plastic pencil is made of hydrophobic resin, the line drawn by the plastic pencil works as a guide for the water-based solution flow.

## Author Contributions

K.N., H.K., M.N. and T.Y. designed this work and K.N. prepared the manuscript. The experiments were carried out by K.N., H.K., T.K., C.U., F.Z., S.R., G.M., Y.H. and K.N., H.K., M.N., T.Y., M.K., K.S., J.D.B., M.J., W.V. have analyzed the results and discussed the manuscript during the preparation. All authors discussed the results and implications and commented on the manuscript at all stages.

## Supplementary Material

Supplementary Information Supplementary Information
