# Supplementary material for: Cellulose Nanofiber Paper as an Ultra Flexible Nonvolatile Memory
Source: Sci Rep. 2014 Jul 2;4:5532. doi: 10.1038/srep05532 (PMC4078308; doi:10.1038/srep05532)
Supplement: Supplementary Information [file srep05532-s1.pdf]

Supplementary Information

Cellulose Nanofiber Paper as an Ultra Flexible Nonvolatile Memory

Kazuki Nagashima,[1] Hirotaka Koga,[1] Umberto Celano,[2] Fuwei Zhuge,[1] Masaki Kanai,[1] Sakon Rahong,[1] Gang Meng,[1] Yong He,[1] Jo De Boeck,[2] Malgorzata Jurczak,[2] Wilfried Vandervorst,[2] Takuya Kitaoka,[3] Masaya Nogi [1] and Takeshi Yanagida [1]

[1]The Institute of Scientific and Industrial Research, Osaka University, 8-1 Mihogaoka Ibaraki, Osaka, 567-0047, Japan

[2] K.U. Leuven at IMEC, Kapeldreef 75 B-3001 Leuven, Belgium

[3]Department of Agro-environmental Sciences, Graduate School of Bioresource and Bioenvironmental Sciences, Kyushu University, Fukuoka, 812-8581, Japan

S1. Characterization of Ag-decorated cellulose nanofibers.

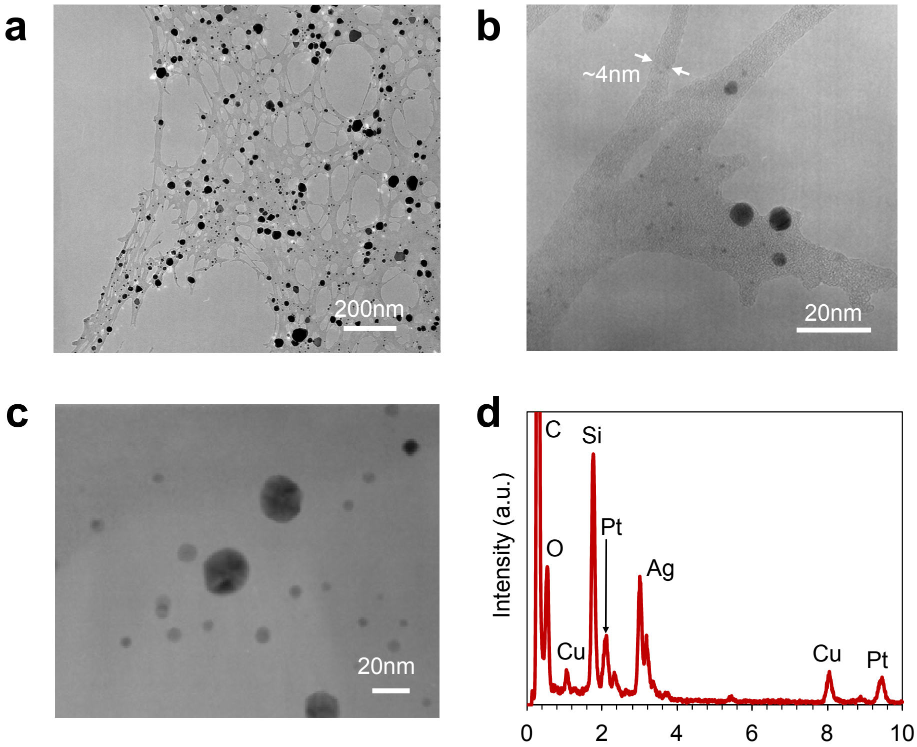

Figure S1 shows (a) low magnification and (b) high magnification transmission electron microscopy (TEM) images of the Ag-decorated cellulose nanofibers diluted by ethanol with 1:500 (Ag-decorated cellulose nanofibers solution : ethanol) ratio. The fiber-like structure with the minimum diameter of about 4 nm was seen. Ag nanoparticles with the diameters of 1-30 nm were decorated on the surface of cellulose nanofibers. (c) TEM image of cross-sectional Ag-decorated cellulose nanofibers paper (CNP). TEM specimen was prepared by making a slice of Ag-decorated CNP device. The close packed structure of CNP without any observable pore was seen. (d) Energy dispersive electron spectroscopy (EDS) of Ag-decorated CNP taken near Pt electrode. Cu peaks are signals from TEM grid.

S2. Analysis of resistive switching in Ag-decorated CNP memory device.

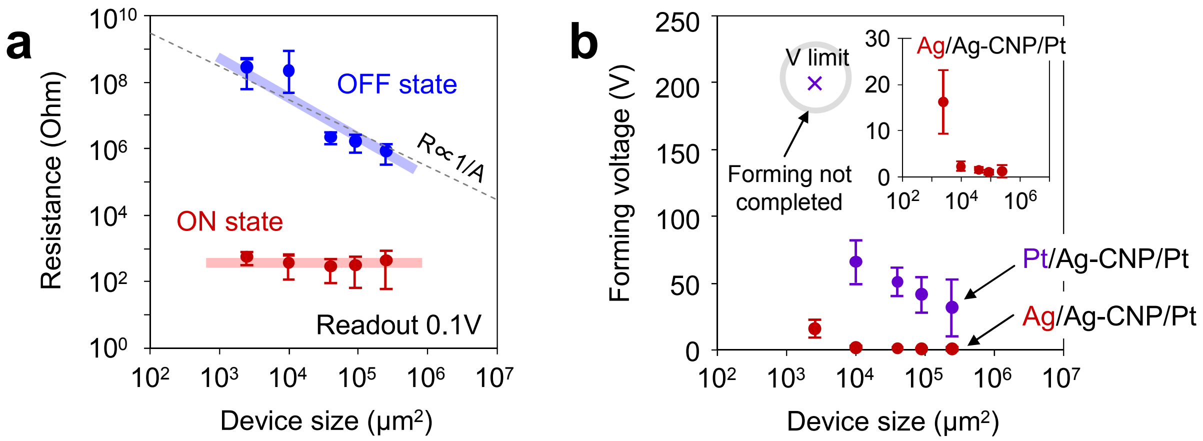

S3. Top electrode material dependence on the nonvolatile resistive switching properties of Ag-decorated CNP device

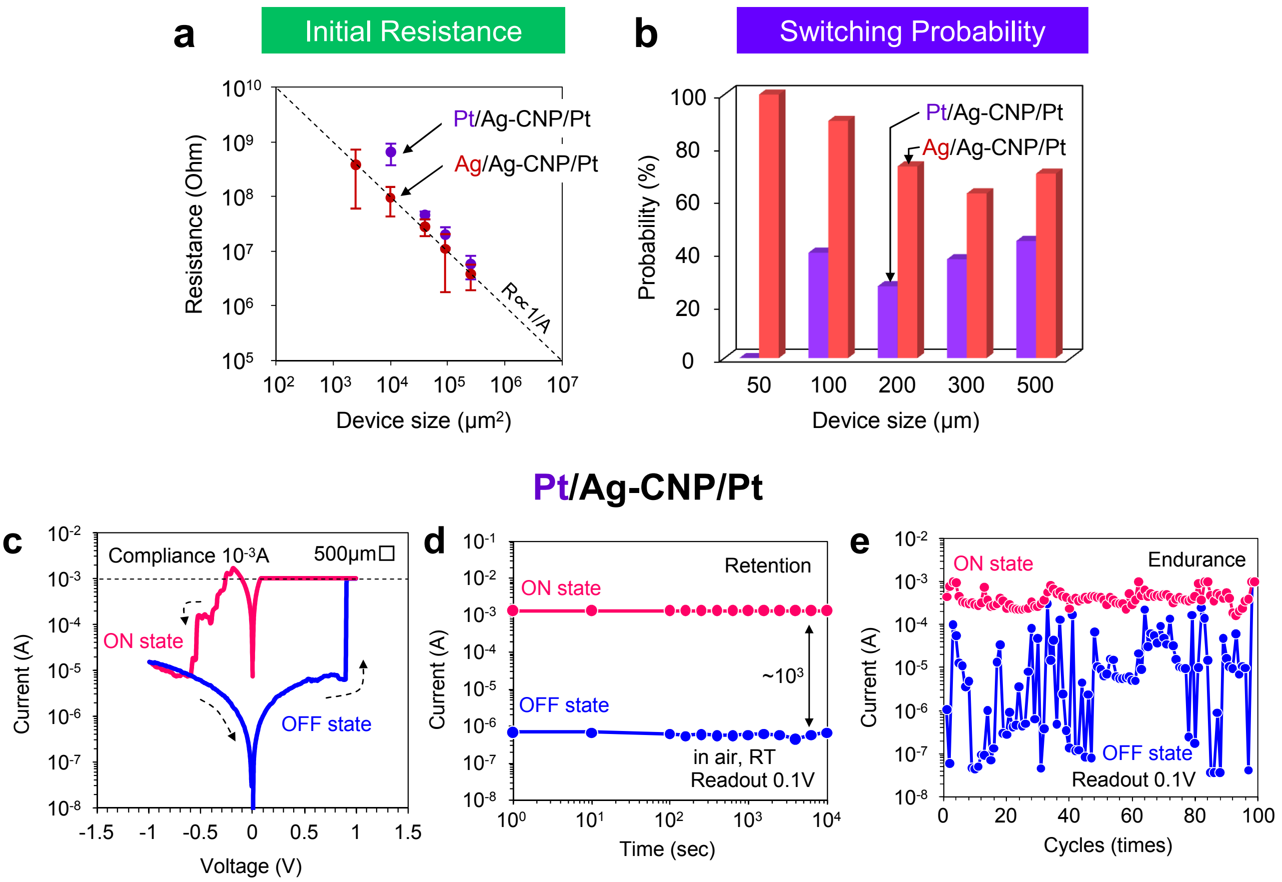

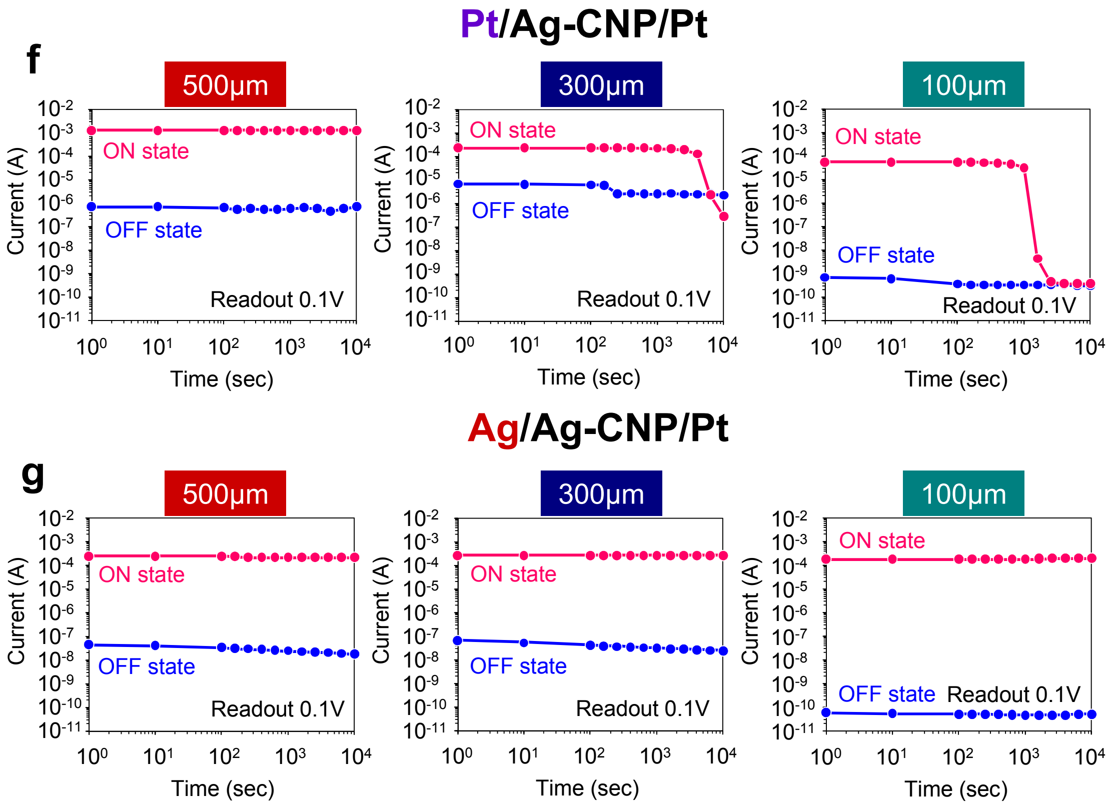

S4. Schematic illustration of resistive switching mechanism and band diagram of Ag-decorated CNP device

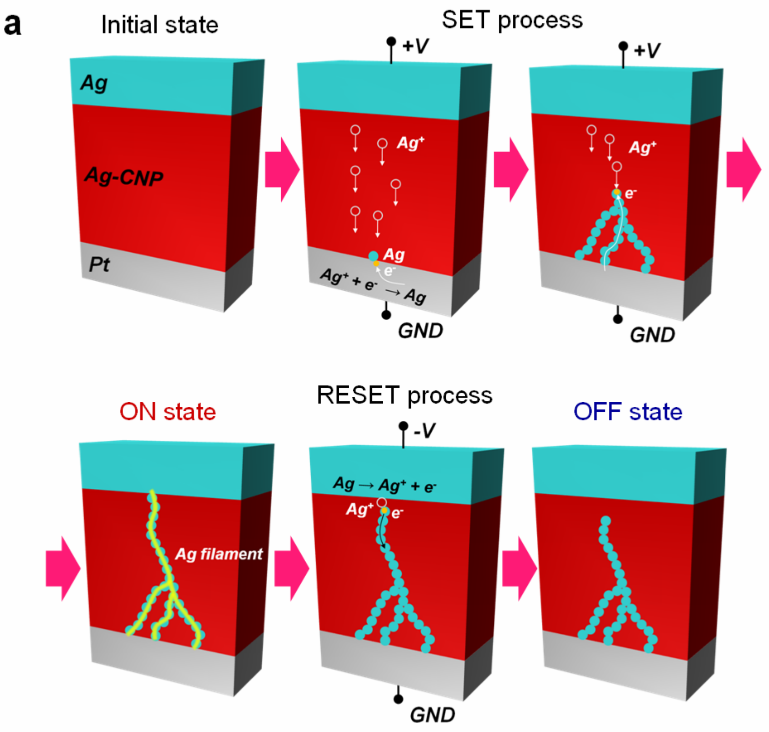

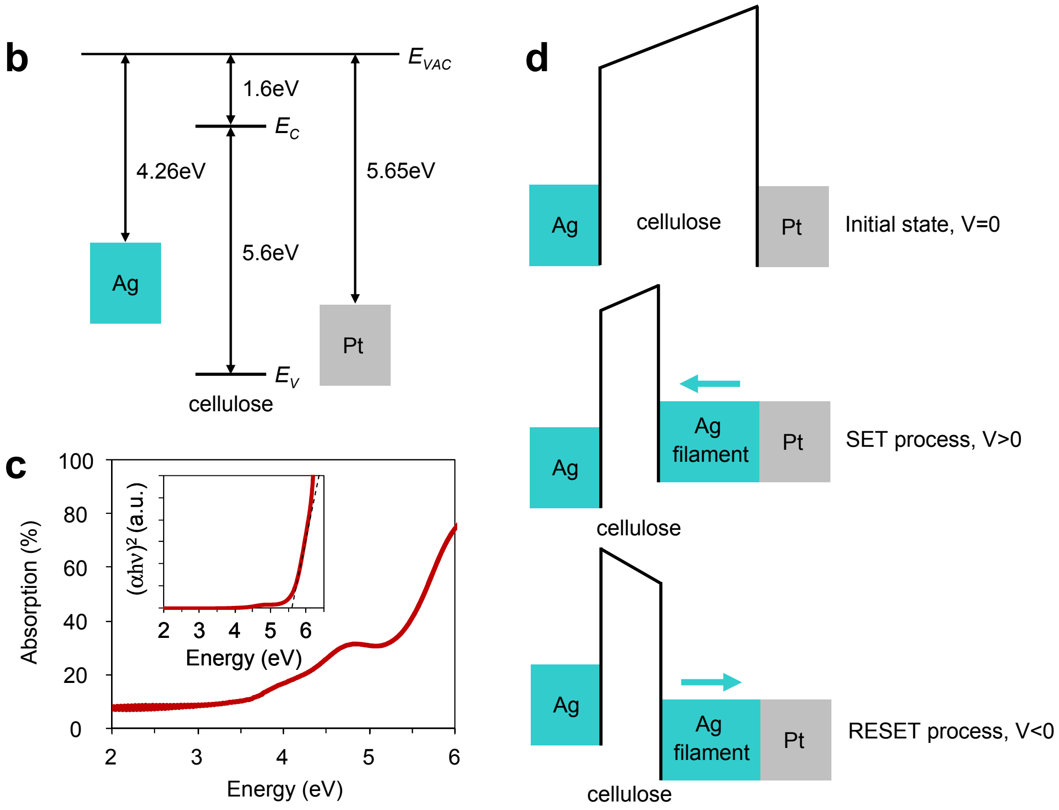

Here we discuss the mechanism for resistive switching in the Ag-decorated CNP devices. Figure S2 (a) shows the device size dependence on the ON and OFF state resistances. The device size was varied from 50×50 μm^2 to 500×500 μm^2. The readout voltage is 0.1 V. The OFF state resistance inversely correlates with the device size. On the other hand, the ON state resistance was independent of the device size, clearly indicating the presence of local conduction paths. In the framework of conventional electrochemical metallization mechanism, the resistive switching can be interpreted in terms of the formation and rupture of Ag conductive filaments. [1-3] First, Ag in the positively biased electrode is ionized to be Ag^+ ions and migrate toward negatively biased Pt counter electrode under the electric field. When the Ag^+ ions reach to the Pt electrode, the Ag^+ ions are reduced by receiving the electrons from the Pt electrode as following reaction; $Ag^+ + e^- \rightarrow Ag$. Such

successive metallization process creates the Ag conductive filaments from the Pt electrode to the Ag electrode. CNP works as the solid electrolyte for Ag^+ ions migration. The resistance state changes to ON state when the Ag filaments are completely bridged between the Ag electrode and the Pt electrode. The rupture of Ag filaments occurs through the oxidization of Ag ($Ag \rightarrow Ag^+ + e^-$) and the migration of Ag^+ ions under the opposite electric field polarity. In such switching model, the ionization and the migration of Ag might be critical for the resistive switching phenomena. Figure S2 (b) shows the effect of top electrode on the forming voltage. In this experiment, we utilized Ag and Pt as the top electrodes and the various device sizes ranging from 50×50 μm^2 to 500×500 μm^2 are examined. Note that the initial resistance values for Ag and Pt top electrodes were comparable (Figure S3 (a)), indicating that the migration of metal during the electrode deposition process is negligible. For both Ag and Pt top electrodes, the forming voltage increased as the device size decreased, which is consistent with the typical electric breakdown phenomenon. [4,5] Remarkably, the forming voltages of the Ag top electrode devices are lower than that of the Pt top electrode devices for all device size. Also the resistive switching with Ag top electrode was more stable than that with Pt top electrode. Figures S3 (b-e) are comparison on (b) switching probability, (c) *I-V* curve, (d) retention and (e) endurance of Pt/Ag-decorated CNP/Pt device. Size dependent data retention is shown for (f) Pt top electrode and (g) Ag top electrode. These results indicate the important role of Ag top electrode for the nonvolatile memory effect in CNP device. Thus, the experimental results highlight that the resistive switching in the Ag-decorated CNP device is based on the formation and rupture of Ag

conductive filaments. The physical picture of discussed resistive switching mechanism is given in figure S4 (a). In addition, the band diagram of present CNP device is shown in figure S4 (b-d). To estimate the energy level of cellulose, we referred the work function of 4.4 eV obtained by ultraviolet photoelectron spectroscopy (UPS). [6] The band gap was estimated to be 5.6 eV by hypothesizing the direct transition of cellulose from UV absorption data as shown in figure S4 (c). UV absorption data was obtained from the TEMPO-oxidized cellulose nanofiber paper with the thickness of ~10 μm. α is absorption coefficient and $h\nu$ is photon energy, respectively.

S5. Electric field simulation for metal nanoparticles embedded CNP.

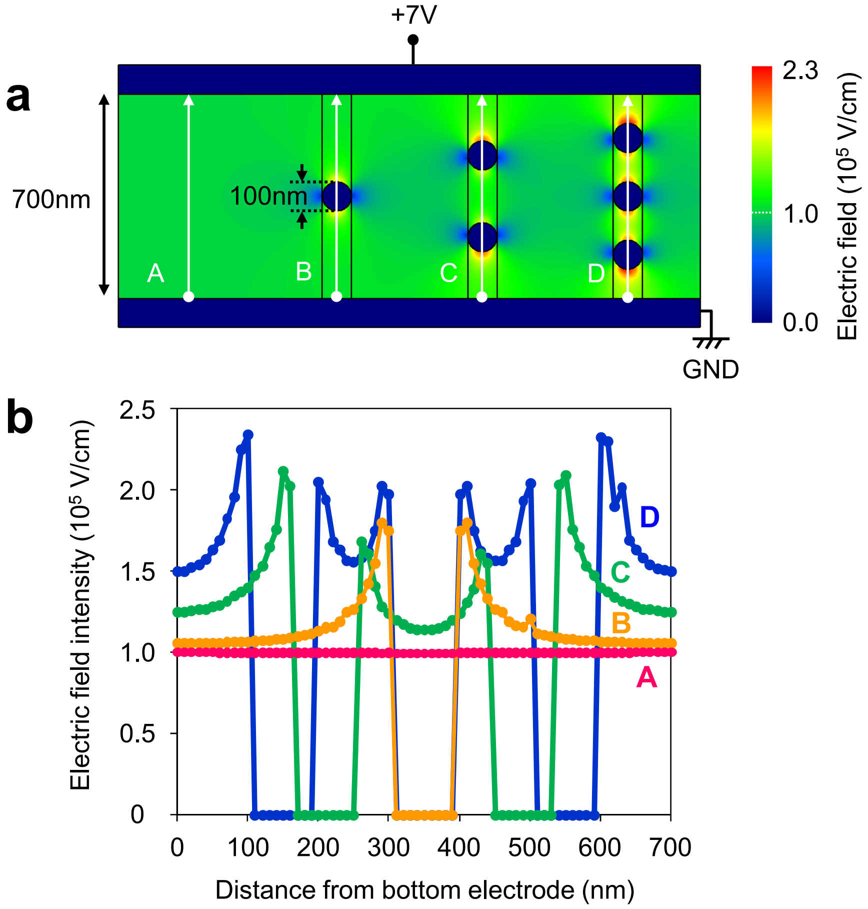

Figure S5 (a) shows the electric field distribution for metal nanoparticles embedded CNP. In this simulation, 2-dimensional electric field simulator, μ-Excel (μ-TEC Co. Ltd.) was utilized. The CNP thickness is 700 nm and the size of metal nanoparticles is 100 nm. For the calculation, the dielectric constant of 7.5 was utilized for CNP. +7 V was applied to the top electrode while the bottom electrode was grounded. We simulate the electric field intensity with varying the number of metal nanoparticles in CNP (A) 0, (B) 1, (C) 2 and (D) 3, respectively. Note that for the case of 3 metal nanoparticles, working distance reduces to 57 % of total CNP thickness, which is less than the substantial working distance of Ag-decorated CNP used in this study. It is clearly seen that the electric field near the metal nanoparticles is enhanced. In addition, the reduction of working

distance enhances the apparent electric field. Figure S5 (b) shows the analyzed data of the electric field intensity along the thickness direction. As discussed above, the electric field is enhanced via 1) the effect of metal nanoparticle edge and 2) the decrease of working distance. However, the maximum electric field intensity of metal nanoparticles embedded CNP is just 2.3 times higher than that of CNP without metal nanoparticles. Therefore, only the enhancement of electric field intensity by the metal nanoparticles cannot explain the extremely low electric field intensity for forming process of Ag-decorated CNP device (1.6×10^{-2} MV/cm).

S6. Selective deposition of Ag-decorated CNP on chemically modified surface.

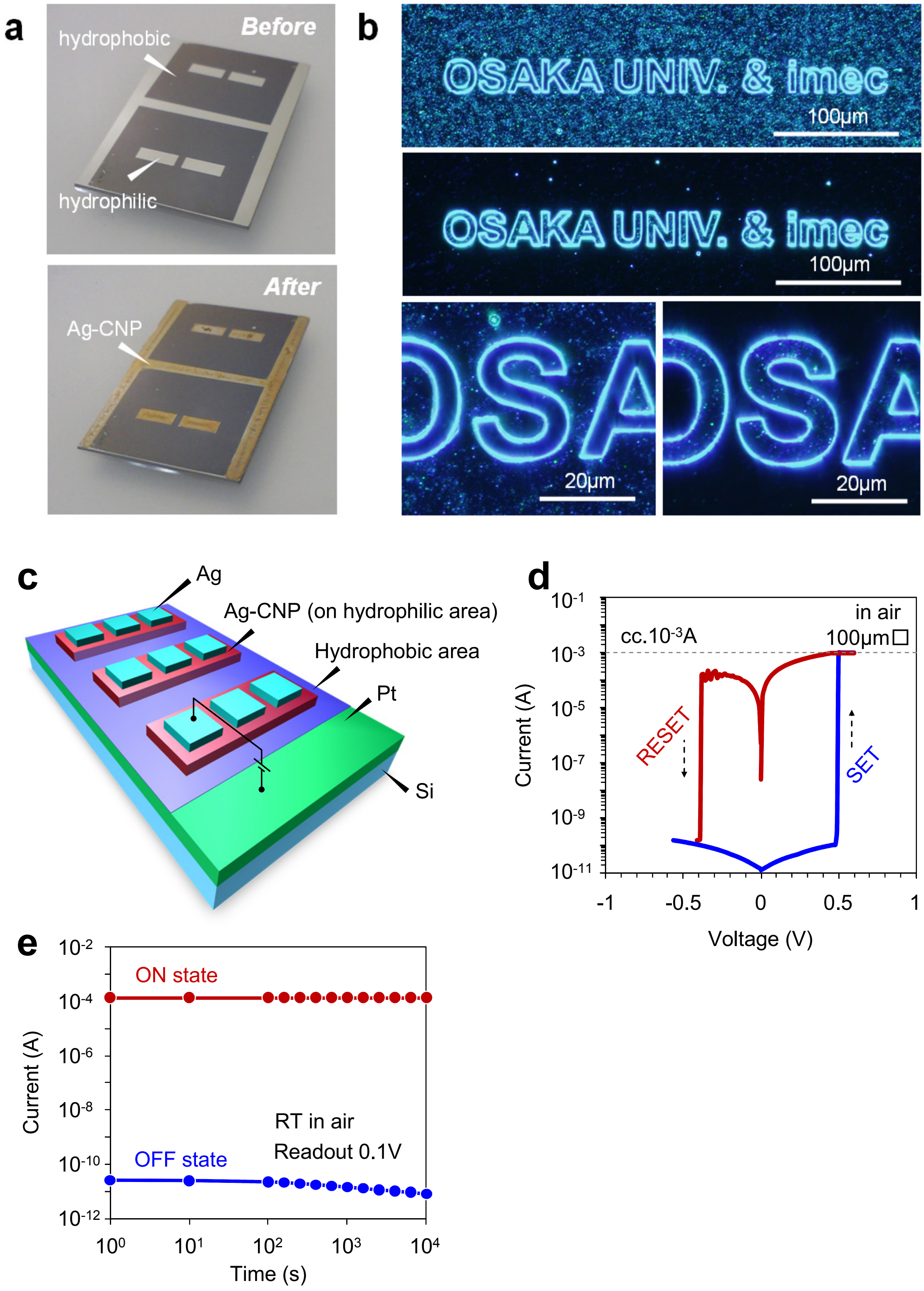

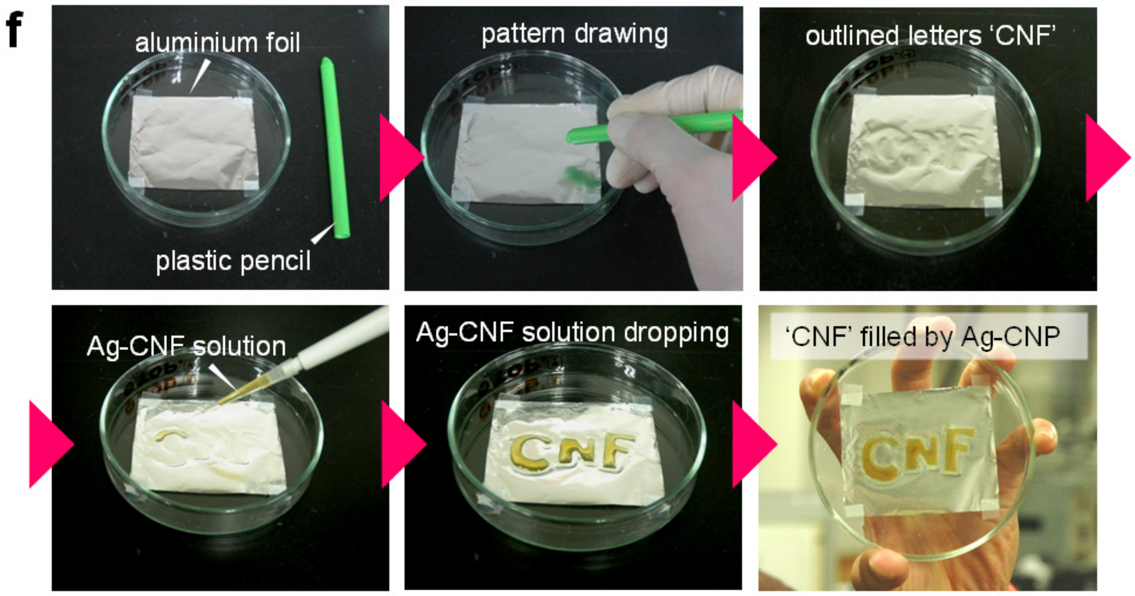

Printing the functional materials on desired surface at low temperature is interesting on the viewpoint of large area, low cost, low energy consumption processing. Here we demonstrate the printability of Ag-decorated CNP devices on desired surface. To create the hydrophilic/hydrophobic pattern, photolithography and 30 kV electron beam (EB) lithography were utilized. Prior to the lithography, 5 nm thick Ti adhesion layer and 100 nm thick Pt bottom electrodes were deposited onto Si. For the photolithography, AZ5206E photo resist (AZ electronic materials) was spin coated at 5500 rpm for 60 s and UV light was irradiated using Xe lamp. For the EB lithography, ZEP520A-7 (ZEON Chemicals) was used as EB resist. After the lithography process, SiO_2 was deposited on the exposed surface area, which was used as a reactive layer for hydrophobic self-assembly monolayer (SAM). Then the substrate was immersed into trichloro-(1H, 1H, 2H, 2H perfluorooctyl)-silane (so-called FDTS) 1 vol% solution in perfluorooctane for 2 hrs, followed by rinsing with perfluorooctane. Subsequently, the resist was lifted-off from the substrate by

sonicating in N,N-dimethylformamide (DMF). Finally we obtained the hydrophilic/hydrophobic pattern where the surface of Pt is hydrophilic and FDTS is hydrophobic. Since the Ag-decorated cellulose nanofibers were suspended in the water, the solution didn't spread to the hydrophobic surface when the solution was dropped onto the hydrophilic surface but not onto the hydrophobic surface, resulting in the selective deposition of the Ag-decorated CNP. Figure S6 (a-b) shows the deposition process of Ag-decorated CNP on lithographically patterned substrate surface. Figure S6 (a) shows photographs before (upper) and after (lower) the deposition of the Ag-decorated CNP on patterned substrate by photolithography. The deposition of Ag-decorated CNP was performed at room temperature. The colored area in the photograph indicates the presence of Ag-decorated CNP. Clearly, Ag-decorated CNP was selectively deposited on the hydrophilic Pt surface. Furthermore, we examine the micrometer-scale patterning of Ag-decorated CNP as shown in the dark field optical microscopy images of figure S6 (b). The surface pattern was designed by EB lithography. The presence of Ag-decorated CNP could be confirmed by the Ag nanoparticles as bright spots in the figures. Clearly, selective deposition of Ag-decorated CNPs could be seen outside (upper) and inside (lower) of the letters 'OSAKA UNIVERSITY & imec'. The width of letter is ~5 μm. Surprisingly, the Ag-decorated CNP was selectively deposited onto hydrophobic surface when the solution was dropped over the whole area. It might be due to the chemical interaction between substrate surface and TEMPO-oxidized cellulose nanofibers. Although the mechanism of this abnormal phenomenon is ambiguous, this methodology offers the micro pattering of the Ag-decorated

CNP with large area, low cost, low energy consumption processing. Thus we successfully demonstrate the printability of Ag-decorated CNP devices at micrometer-scale resolution.

Then the resistive switching effect was confirmed in the selectively deposited Ag-decorated CNP. Figure S6 (c) shows the schematic illustration of Ag-decorated CNP device selectively deposited on hydrophilic surface. Ag/Ag-decorated CNP/Pt structure was fabricated by deposition of Ag top electrode. Figure S6 (d) and (e) show the *I-V* characteristics and data retention of the device. The measurement was performed at room temperature in air condition. The thickness of Ag-decorated CNP was 750 nm. The compliance current of 10^{-3} A was applied. The clear resistive switching and the good retention properties were confirmed.

Next we examined the feasibility of hand-drawing process for the selective deposition of Ag-decorated CNP on the arbitrarily designed surface. Figure S6 (f) shows the fabrication procedure of the arbitrarily designed Ag-decorated CNP. We utilize an aluminium foil as a substrate. The surface pattern was drawn by using a plastic pencil (Coupy pencil; SAKURA Color Product Corp.). Since the plastic pencil is made of hydrophobic resin, the line drawn by the plastic pencil works as the guide for flowing the Ag-decorated CNP solution. As shown in figure S6 (f), we successfully deposited the Ag-decorated CNP on the arbitrarily designed characters ‘CNF’. Thus these results offer the feasibility of Ag-decorated CNP memory by low cost, low energy consumption printing processing.

S7. Micro structure of Ag-decorated CNP on aluminium foil.

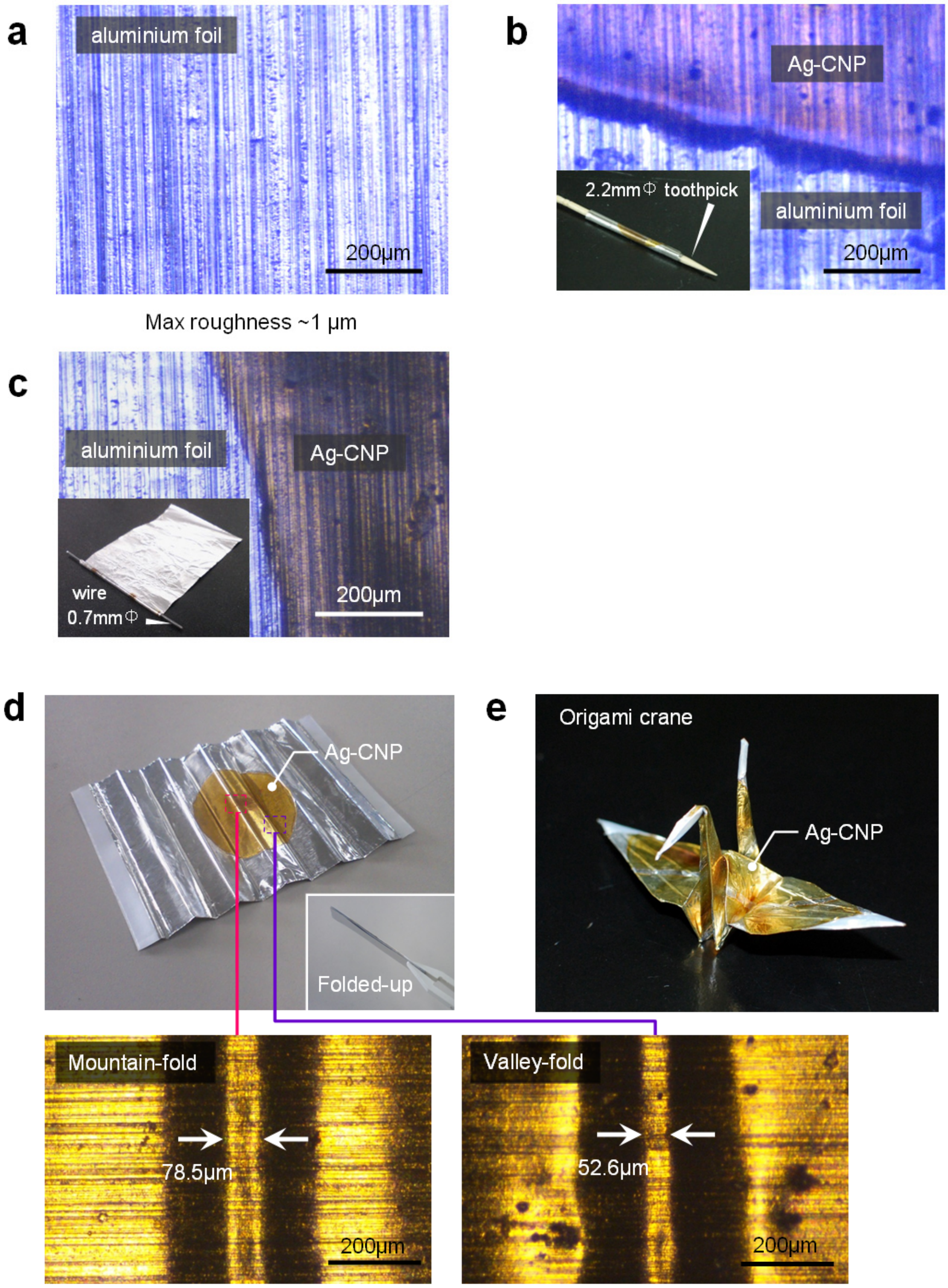

Figure S7 shows the micro structure of Ag-decorated CNP on aluminium foil observed by optical microscopy. (a) The aluminium foil before Ag-decorated CNP deposition. The maximum height difference between top and valley was about 1 μm. (b) The micro structure of Ag-decorated CNP near the edge, which was taken after wrapped around a toothpick (ϕ=2.2 mm) as shown in the inset. The thickness of Ag-decorated CNP was 560 nm. (c) The micro structure of Ag-decorated CNP near the edge, which was taken after wrapped around a metal wire (ϕ=0.7 mm) as shown in the inset. None of any cracks and exfoliation was seen for all over the sheet, indicating the mechanical flexibility of Ag-decorated CNP.

To further investigate the flexible performance of the Ag-decorated CNP, we examined the folding tests as shown in figure S7 (d). Firstly, we prepare the 1 μm thick Ag-decorated CNP on aluminium foil. Then we fold up the aluminium foil as shown in inset of figure S7 (d). After expanding, we perform the optical microscopy observations. Lower figure S7 (d) shows the optical microscopy images taken at the mountain-fold area and the valley-fold area. The surface cannot be back to totally flat after folding process due to the shape memory effect of aluminium foil. Therefore the image near the valley looks dark since the optical light on the surface reflect to the inclined angle. There were no observable cracks and exfoliations at the folded area. Here we estimate the curvature radius for the folding test. In the optical microscopy images, the widths of folded area are 78.5 μm for mountain-fold and 52.6 μm for valley-fold, respectively. Note that the larger width for mountain-fold area is reasonable by taking into account the thickness of Ag-decorated CNP and aluminium foil.

From the widths, the curvature radii can be roughly calculated to be ~25.0 μm (mountain-fold) and ~16.8 μm (valley-fold). As such, the structure of Ag-decorated CNP is mechanically sustainable for both tensile and compressive strain at least down to several tens micrometers of curvature radius.

Taking the advantage of foldability, here we demonstrate an arbitrarily formed Ag-decorated CNP. Figure S7 (e) shows the photograph of ‘origami crane’ fabricated by folding the Ag-decorated CNP on aluminium foil. Although the sheet was folded many times during the origami process, the cracks and the exfoliation were not observed. Thus we successfully demonstrated the excellent mechanical flexibility of the Ag-decorated CNP for the flexible resistive switching memory devices.

References

1. Waser, R. & Aono, M. Nanoionics-based resistive switching memories. *Nat. Mater.* **6**, 833-840 (2007).
2. Waser, R., Dittmann, R., Staikov, G. & Szot, K. Redox-based resistive switching memories –nanoionic mechanisms, prospects, and challenges. *Adv. Mater.* **21**, 2632-2663 (2009).
3. Valov, I. *et al.* Nanobatteries in redox-based resistive switches require extension of memristor theory. *Nat. Commun.* **4**:1771 DOI: 10.1038/ncomms2784, 1-9 (2013).
4. Satoh, T. & Tanaka R. Area effect and distance effect of transformer oil insulation. *Aich Denki Giho* **28**, 12-17 (2007).
5. Schmalhorst, J. *et al.* Evolution of the dielectric breakdown in Co/Al_2O_3/Co junctions by annealing. *J. Appl. Phys.* **89**, 586-589 (2001).
6. Dahle, S., Meuthen, J., Viöl, W. & Maus-Friedrichs, W. Adsorption of silver on cellobiose and cellulose studied with MIES, UPS, XPS and AFM. *Cellulose* **20**, 2469-2480 (2013).
